# Evaluation of berberine pellet effect on clinical recovery time in COVID-19 outpatients: A pilot clinical trial

**DOI:** 10.22038/AJP.2022.21539

**Published:** 2023

**Authors:** Soodabeh Omidvar Tehrani, Mahboobeh Ghasemzadeh Rahbardar, Kamran Shoorgashti, Mohammad Javad Dehghan Nayeri, Amir Hooshang Mohammadpour, Hossein Hosseinzadeh

**Affiliations:** 1 *School of Pharmacy, Mashhad University of Medical Sciences, Mashhad, Iran*; 2 *Pharmaceutical Research Center, Pharmaceutical Technology Institute, Mashhad University of Medical Sciences, Mashhad, Iran *; 3 *Infection Control & Hand Hygiene Research Center, Mashhad University of Medical Sciences, Mashhad, Iran*; 4 *Department of Clinical Pharmacy, School of Pharmacy, Mashhad University of Medical Sciences, Mashhad, Iran*; 5 *Department of Pharmacodynamics and Toxicology, School of Pharmacy, Mashhad University of Medical Sciences, Mashhad, Iran*

**Keywords:** Clinical trial, Berberis vulgaris, Barberry, Coronavirus disease, Lymphopenia, Antiviral

## Abstract

**Objective:**

Severe disease onset of COVID-19 may result in alveolar injury and respiratory failure. Apoptosis and inflammation are the main causes of respiratory distress syndrome. Berberine is used in medicine as an analgesic, anti-asthmatic, anti-inflammatory, and antiviral. In the current investigation, the effect of berberine on COVID-19 outpatients was studied.

**Materials and Methods:**

The present clinical trial was performed on 40 outpatients who were randomly assigned to berberine (300 mg, TID, 2 weeks) (n=19) or placebo groups (n=21). Both groups received standard therapy and they were monitored on days 3, 7, and 14 after the beginning of the therapy for clinical symptoms’ improvement, quantitative CRP, lymphopenia, CBC, and SpO_2_. The severity and frequency of these symptoms and the level of the parameters were statistically compared between the two groups.

**Results:**

On days (0, 3, 7, and 14, there was no significant difference between the berberine and placebo groups in the improvement of clinical symptoms (cough, shortness of breath, nausea, loss of smell and taste, diarrhea, dizziness, sore throat, stomachache, body aches, and body temperature), quantitative CRP, lymphopenia, WBC, neutrophils, platelets, or SpO_2_.

**Conclusion:**

Berberine (300 mg, TID, two weeks) is ineffective in treating COVID-19. More research with a larger sample size is needed to investigate different berberine dosages in other pharmaceutical formulations.

## Introduction

Since the outbreak of the coronavirus disease 2019 (2019-nCoV) in Wuhan, China in December 2019, the virus has spread rapidly throughout the world and has become a major global health concern (Babaei et al., 2020; Rameshrad et al., 2020). On February 11, 2020, the World Health Organization (WHO) announced a new name for the epidemic caused by nCoV-2019, COVID-19 (Gorbalenya et al., 2020). On January 30, 2020, the WHO announced the outbreak of COVID-19 as the sixth international public health emergency, after influenza A virus subtype H1N1 (2009), polio (2014), Ebola in West Africa (2014), Zika (2016), and Ebola in the Democratic Republic of the Congo (2019) (Yoo, 2020).

In general, COVID-19 can be lethal, with a mortality rate of 2%. The clinical spectrum of COVID-19 varies from asymptomatic to respiratory failure which requires mechanical ventilation and hospitalization in the intensive care unit (ICU). Some patients also have systemic clinical manifestations of sepsis, septic shock, and organ dysfunction syndromes. Severe disease onset may result in extensive alveolar injury and progressive respiratory failure (Chan et al., 2020; Huang et al., 2020). Therefore, the virus was named Acute Respiratory Syndrome of Coronavirus 2 (SARS-CoV-2) by the International Committee for Classification of Viruses (Babaei et al., 2021; Fung et al., 2020). In this regard, in one of the first reports related to this disease, it was shown that patients suffer from fever, weakness, dry cough, and shortness of breath. Chest computerized tomography (CT) scans in all cases showed samples with abnormal findings, and about one-third of these individuals required admission to the ICU (Huang et al., 2020). Treatments are based on patients' symptoms, and oxygen therapy is one of the main therapeutic interventions for patients with severe infections. Mechanical ventilation may also be required in cases of oxygen-resistant respiratory failure.

The pathophysiology of SARS-CoV-2 is not well understood, but it is likely to be similar to SARS-CoV. Acute lung damage due to SARS-CoV infection is mainly due to inflammation caused by virus replication (García, 2020). The initial onset of rapid viral replication may lead to widespread apoptosis in alveolar and endothelial epithelial cells and vascular leakage, which results in the secretion of pro-inflammatory cytokines and chemokines (Darif et al., 2021). Thus, similar to SARS-CoV infection, SARS-CoV-2 infection increases the secretion of interleukin 1 beta (IL-1β), interferon-c (IFN-c), human interferon-inducible protein 10 (IP-10), monocyte chemoattractant protein-1 (MCP-1), IL-4, and IL-10. Furthermore, a comparison between patients admitted to the ICU and patients admitted to normal treatment units showed that patients admitted to the ICU had higher plasma levels of IL-2, IL-7, IL-10, granulocyte colony-stimulating factor (GCSF), IP-10, MCP-1, macrophage inflammatory protein-1 alpha (MIP-1-α), and tumor necrosis factor-alpha (TNF-α), and it was suggested that cytokine storms may be associated with disease severity (Huang et al., 2020). Considering that uncontrolled pulmonary infection is probably the leading cause of death in SARS-CoV-2 infection, the use of herbal compounds that have strong anti-inflammatory and antimicrobial roles may be effective (Boozari and Hosseinzadeh, 2021; Brendler et al., 2021). 

Berberine is an isoquinoline alkaloid purified from barberry (*Berberis vulgaris*) and used to treat microbial diarrhea (Imanshahidi and Hosseinzadeh, 2008; Imenshahidi and Hosseinzadeh, 2016). Numerous studies have reported the antiviral effects of berberine, including influenza virus (Wu et al., 2011), influenza H1N1 (Yan et al., 2018), enterovirus 71 (Wang et al., 2017), Chicago virus (Varghese et al., 2016), and herpes simplex virus (Dkhil and Al-Quraishy, 2014). The anti-inflammatory property of berberine is induced by 5' adenosine monophosphate-activated protein kinase (AMPK) activation, nuclear factor-kappa B (NF-κB) inhibition, and activator protein-1 (AP-1) pathway inhibition (Wang et al., 2017). The inhibition of these pathways by berberine plays an important role in inflammation and carcinogenesis, leading to decreased expression of cytokines including TNF-α, IL-1β, IL-6, MCP-1, inducible nitric oxide synthase (iNOS), and cyclooxygenase-2 (COX-2) (Zou et al., 2017). Berberine has also been reported to have an antagonistic effect on muscarinic receptors, and its mechanism of action includes activation of the adenylate cyclase/cyclic adenosine monophosphate (cAMP) pathways (Sánchez‐Mendoza et al., 2008). Numerous studies have shown the positive effects of berberine on pulmonary disorders and respiratory distress syndrome. For example, the results of a study showed that berberine reduces airway inflammation by inhibiting the NF-κB signaling pathway in albumin-induced asthmatic rats (Li et al., 2016). Another study found that berberine reduced endothelial glycocalyx degradation and increased glycocalyx repair in lipopolysaccharide-induced acute respiratory distress syndrome (ARDS) (Huang et al., 2018). 

Given that inflammation is important in the pathophysiology of COVID-19 and that berberine has antiviral, anti-allergy, anti-inflammatory, and antioxidant properties, it is possible that berberine can alleviate COVID-19 symptoms. So far, no clinical trials have been conducted in this area. Thus, our team decided to investigate the effect of berberine (300 mg, three times a day, 2 weeks) besides standard therapy on the recovery of COVID-19 outpatients who were monitored on days 3, 7, and 14 after beginning the therapy for clinical symptom improvement, quantitative C-reactive protein (CRP), lymphopenia, complete blood count (CBC), and arterial oxygen level (SpO_2_).

## Materials and Methods

**Determining the purity percentage of berberine commercial sample**

**Ultraviolet (UV) method**

First, 20 mg of Sigma standard berberine with an 86% purity and a pharmaceutical berberine sample with an unknown purity were weighed and volumized before being filtered in a 500 mL balloon with distilled water (40 ppm). Then from concentrations of 10 ppm, concentrations of 8 and 7 ppm were prepared and the absorptions were then read by Shimadzu UV Visible spectrophotometer. The purity percentage of the commercial sample was calculated by comparing its absorption rate to that of the standard sample.

**High-performance liquid chromatography (HPLC) method**

To begin, 10 mg of each sample, the standard reference with confirmatory purity of 86% and the pharmaceutical sample with unknown purity, were weighed and volumized in a 100 mL balloon with HPLC grade methanol (concentration 100 ppm). The solutions were then mixed with 5 mL of methanol to achieve a concentration of 50 ppm. The obtained samples were analyzed using the HPLC method, and the purity percentage of the commercial sample was determined by comparing the area below the Chromatogram diagram.

**Pellet making process**

The pellets were prepared using the extrusion-spheronization technique as follows:

The solid components of the pellets, which included 60% berberine, 35% microcrystalline cellulose, and 5% cross carmellose, were thoroughly mixed to produce a homogeneous mixture.

Granulation: A uniform wet mass of suitable consistency was obtained by adding distilled water dropwise during granulation and mixing. 6 ml of water was required for 15 g of powder.

Extrusion: The wet mass from the extruder with a die pore diameter of 1 mm and a speed of 100 rpm was passed through this stage to obtain suitable extrudates.

Spheronization: The extrudates were placed in a spheronizer at 1000 rpm for 6 min to produce spherical pellets. 

Drying: Wet pellets were dried in an oven at 40°C for 4 hr.

**Patients**

**Figure 1:**
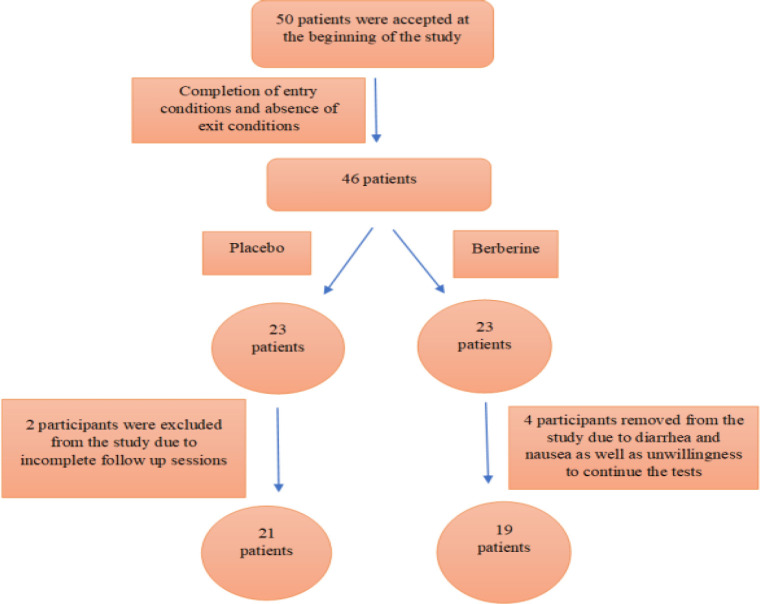
A consort flowchart of participants

This study was conducted on 40 outpatients with COVID-19 at the outpatient clinic of Mashhad University of Medical Sciences from May 6 to December 16, 2020, considering the inclusion and exclusion criteria (Figure. 1). The protocol was approved by the Iranian Committee of Ethics in Human Research (Registration number IR.MUMS.REC.1399.053), approved May 2020, and the trial was registered (ClinicalTrials.gov number, IRCT20081019001369N2).

**Inclusion criteria**

People diagnosed with coronavirus based on the following criteria could enter the present trial:

-clinical symptoms (fever, cough, and myalgia), laboratory symptoms (lymphopenia and increase in C-reactive protein (CRP)), diagnosis of the infectious disease specialist, not meeting the exclusion criteria, having the indication of home quarantine and medication use on an outpatient basis.

**Exclusion criteria**

Patients with any of the following criteria were excluded from the trial:

- Having a catheter or undergoing chemotherapy 

- Taking cytotoxic drugs or corticosteroids

- Pregnant and lactating patients

- Diabetic patients

- Patients with severe renal insufficiency or liver failure Child-Pugh B, C

- Patients with normal cell count or normal CRP

- Patients with autoimmune diseases (rheumatoid arthritis, systemic lupus erythematosus, etc.)

- Patients with inflammatory bowel disease

- Patients with drug-related contraindications

- Patients with other sites of infection at the same time

- Patients participating in other clinical trials

- Patients requiring hospitalization according to clinical and laboratory criteria.

**Study design**

The study included forty patients (aged 18–65 years old) with a coronavirus diagnosis based on clinical and laboratory symptoms, as well as an infectious disease specialist, who had indications for taking the medicines on an outpatient basis and met the inclusion criteria. According to the ethical consultation of the clinic, patients voluntarily signed the consent designed for this trial. 

All patients were randomly assigned to one of two groups: treatment (n=19) or placebo (n=21) and received standard COVID-19 outpatient therapy based on the Iranian national COVID-19 treatment protocol and best practice guidelines at the time. Berberine (300 mg, three times a day for two weeks) and a placebo (Avicel® microcrystalline cellulose, 400 mg, three times a day, 2 weeks) were administered to patients in a double-blind and randomized manner.

The patients referred were monitored at intervals of 3 days, 1 week, and 2 weeks after receiving medication or placebo and recovery time of lymphopenia (<1100 cells/mm^3^), CRP (>6 mg/L) normalization time course, and amelioration time of clinical symptoms (cough, shortness of breath, nausea, loss of smell and taste, runny or stuffy nose, diarrhea, dizziness, sore throat, stomachache, body aches, and body temperature).

Medical symptoms were assessed by a clinical pharmacist blinded to the treatment allocation. According to each criterion, clinical symptoms were classified as mild, moderate, or severe. A standardized questionnaire was used at the start of the study, on days 3 and 7, and at the end of the treatment period.

**Primary endpoint**

The primary goal was to determine whether berberine reduced the recovery time of laboratory and clinical symptoms.

**Secondary endpoint**

The effect of berberine on the severity of clinical symptoms besides laboratory symptoms.

**Randomization and blinding**

A medical student tagged placebos and matched them to the berberine in terms of color, size, and packaging. All participants, including nurses, researchers, physicians, and patients were unaware of the code written on the capsule packaging. Randomization was conducted using computer-generated random numbers. The researchers and physicians were not told about the randomization and allocation until after the analyses were completed. In the hospital, a trained nurse was in charge of randomizing patients and assigning them to two groups.

**Statistical analysis**

For reporting quantitative and qualitative variables, mean ± standard deviation and grading (mild, moderate, and severe) were used, respectively.

The Shapiro-Wilk test was used to distinguish between normally and non-normally distributed quantitative data, which was then compared between the groups using independent sample t-tests and Mann-Whitney tests.

The chi-square test was used to compare qualitative variables between the two groups. Repeated measures ANOVA was used to compare laboratory findings such as CRP, leukocytes, lymphocytes, and SpO_2_ between the two groups on different days of 0, 3, 7, and 14. SPSS software (version 21, SPSS Inc., Chicago, IL, USA) was used for statistical analysis, and a p<0.05 was considered significant.

## Results

**Berberine spectra in distilled water**

**Figure 2:**
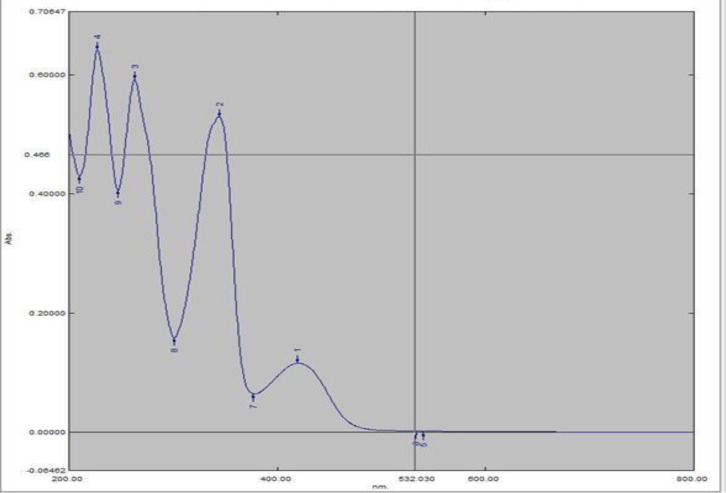
The absorption spectrum of berberine solution in distilled water (range of 800-200 nm)

Berberine spectra were recorded in distilled water medium and a UV range of three λmax (230, 266, and 349 nm). These three wavelengths were selected for the validation process (Figure 2). 

**Study population**

40 patients with confirmed COVID-19 disease were studied. Patients were randomly distributed into the treatment and placebo (control) groups, with 21 in the placebo and 19 in the treatment groups. The statistical analysis discovered that the demographics and severity of disease between these groups of individuals were similar. The average age was 44.36±11.75 years in the treatment group and 44.52±12.61 years in the control group. In terms of gender, 16 were female and 24 were male, with 57.89 percent and 61.91 percent of males in the treatment and placebo groups, respectively.

**Comparison of clinical symptoms in the treatment and placebo groups on different days**

The difference in coughing between the treatment and placebo groups on days 0 (p=0.965), 3 (p=0.739), 7 (p=0.482), and 14 (p=0.660) was not statistically significant (Table 1).

On days 0 (p=0.670), 3 (p=0.282), 7 (p=0.561), and 14 (p=0.970) (Table 2) the difference in the frequency of shortness of breath between the treatment and placebo groups was not statistically significant.

The difference in the frequency of nausea between the treatment and placebo groups on days 0 (p=0.987), 3 (p=0.983), 7 (p=0.841), and 14 (p=0.207) was not statistically significant (Table 3). 

On days 0 (p=0.206), 3 (p=0.395), 7, (p=0.386), and 14 (p=0.514) (Table 4) there was no statistically significant difference in the incidence of loss of smell the between treatment and placebo groups.

**Table 1:** Comparison of the frequency of cough between the treatment and placebo groups on different days

**Time (day)**	**Grade**	**Treatment (%)**	**Placebo (%)**	**p-value (Chi-square test)**
0 (n=40)	0	10.52 (n=2)	14.2 (n=3)	0.965
	1	26.31 (n=5)	28.57 (n=6)	
	2	47.3 (n=9)	47.6 (n=10)	
	3	10.52 (n=2)	4.76 (n=1)	
	4	5.2 (n=1)	4.76 (n=1)	
3 (n=40)	0	15.7 (n=3)	9.52 (n=2)	0.739
	1	57.89 (n=11)	57.14 (n=12)	
	2	23.8 (n=5)	15.78 (n=3)	
	3	5.26 (n=1)	9.52 (n=2)	
	4	5.26 (n=1)	0	
7 (n=40)	0	26.31 (n=5)	33.33 (n=7)	0.482
	1	52.63 (n=10)	38.09 (n=8)	
	2	15.78 (n=3)	28.57 (n=6)	
	3	0	0	
	4	5.26 (n=1)	0	
14 (n=40)	0	57.89 (n=11)	80.95 (n=14)	0.660
	1	36.84 (n=7)	14.28 (n=5)	
	2	5.26 (n=1)	4.76 (n=1)	
	3	0	4.76 (n=1)	
	4	0	0	

**Table 2:** Comparison of the frequency of shortness of breath between the treatment and placebo groups on different days

**Time (day)**	**Grade**	**Treatment (%)**	**Placebo (%)**	**p-value (Chi-square test)**
0 (n=40)	0	26.31 (n=5)	28.57 (n=6)	0.670
	1	42.10 (n=8)	42.85 (n=9)	
	2	26.3 (n=5)	14.28 (n=3)	
	3	5.26 (n=1)	14.28 (n=3)	
	4	0	0	
3 (n=40)	0	42.1 (n=8)	47.6 (n=10)	0.282
	1	36.84 (n=7)	19.04 (n=4)	
	2	15.78 (n=3)	9.52 (n=2)	
	3	5.26 (n=1)	23.8 (n=5)	
	4	0	0	
7 (n=40)	0	63.15 (n=12)	80.95 (n=17)	0.561
	1	26.31 (n=5)	9.52 (n=2)	
	2	5.26 (n=1)	4.76 (n=1)	
	3	5.26 (n=1)	4.76 (n=1)	
	4	0	0	
14 (n=39)	0	77.77 (n=14)	80.95 (n=17)	0.970
	1	16.66 (n=3)	14.28 (n=3)	
	2	5.55 (n=1)	4.76 (n=1)	
	3	0	0	
	4	0	0	

Grades: 0: No shortness of breath; 1: Mild; 2: Moderate; 3: Severe; 4: Very severe

**Table 3:** Comparison of the frequency of nausea between the treatment and placebo groups on different days

**Time (day)**	**Grade**	**Treatment (%)**	**Placebo (%)**	**p-value (Chi-square test)**
0 (n=40)	0	63.15 (n=12)	61.9 (n=13)	0.987
	1	21.05 (n=4)	19.04 (n=4)	
	2	10.52 (n=2)	14.28 (n=3)	
	3	5.26 (n=1)	4.76 (n=1)	
3 (n=40)	0	73.68 (n=14)	76.19 (n=16)	0.983
	1	10.52 (n=2)	9.52 (n=2)	
	2	15.78 (n=3)	14.28 (n=3)	
	3	0	0	
7 (n=40)	0	73.68 (n=14)	66.66 (n=14)	0.841
	1	21.05 (n=4)	23.8 (n=5)	
	2	5.26 (n=1)	9.52 (n=2)	
	3	0	0	
14 (n=40)	0	89.47 (n=17)	95.23 (n=20)	0.207
	1	0	4.76 (n=1)	
	2	10.52 (n=2)	0	
	3	0	0	

Grades: 0: no nausea; 1: mild; 2: moderate; 3: severe

**Table 4:** Comparison of loss of smell between the treatment and placebo groups on different days

**Time (day)**	**Grade**	**Treatment (%)**	**Placebo (%)**	**p-value (Chi-square test)**
0 (n=40)	0	52.63 (n=10)	28.57 (n=6)	0.206
	1	21.05 (n=4)	19.04 (n=4)	
	2	26.31 (n=5)	52.38 (n=11)	
3 (n=40)	0	52.63 (n=10)	33.33 (n=7)	0.395
	1	26.84 (n=5)	28.57 (n=6)	
	2	21.05 (n=4)	38.09 (n=8)	
7 (n=40)	0	52.63 (n=10)	47.61 (n=10)	0.386
	1	31.57 (n=6)	19.04 (n=4)	
	2	14.28 (n=3)	33.33 (n=7)	
14 (n=34)	0	68.42 (n=13)	61.9 (n=13)	0.514
	1	21.05 (n=4)	21.05 (n=4)	
	2	10.52 (n=2)	10.52 (n=2)	

Grades: 0= complete sense of smell; 1 = relative change in sense of smell, 2 = no sense of smell

There was no statistically significant difference in the incidence of loss of taste between the treatment and placebo groups on days 0 (p=0.724), 3 (p=0.801), 7, (p=0.757), and 14 (p=0.669) (Table 5).

According to the obtained data (Table 5), on days 0 (p=0.510), 3, (p=0.386), 7, (p=0.538), and 14 (p=0.538), there was no statistically significant difference in the 

incidence of runny nose between the treatment and placebo groups.

There was no statistically significant difference in the frequency of diarrhea between the treatment and placebo groups on days 0 (p=0.637), 3, (p=0.543), 7, (p=0.556), and 14 (p=0.475) (Table 6).

As the obtained data revealed there was no significant difference in the incidence of dizziness between the treatment and placebo groups on days 0 (p=0.913), 3, (p=0.512), 7, (p=0.380), and 14 (p=0.564) (Table 6).

**Table 5:** Comparison of loss of taste and runny nose between the treatment and placebo groups on different days

**Factors**	**Time (day)**	**Grade**	**Treatment (%)**	**Placebo (%)**	**p-value (Chi-square test)**
**Loss of taste**	0 (n=40)	0	52.63 (n=10)	42.85 (n=9)	0.724
		1	21.05 (n=4)	19.04 (n=4)	
		2	26.31 (n=8)	38.09 (n=8)	
	3 (n=40)	0	52.63 (n=10)	42.85 (n=9)	0.801
		1	26.31 (n=5)	28.57 (n=6)	
		2	21.05 (n=4)	28.57 (n=6)	
	7 (n=40)	0	57.89 (n=11)	57.14 (n=12)	0.757
		1	26.31 (n=5)	19.04 (n=4)	
		2	15.7 (n=3)	23.08 (n=5)	
	14 (n=40)	0	37.68 (n=14)	71.42 (n=14)	0.669
		1	15.78 (n=3)	15.78 (n=3)	
		2	10.52 (n=2)	10.52 (n=2)	
Runny nose	0 (n=40)	0	89.74 (n=17)	90.47 (n=19)	0.510
		1	10.52 (n=2)	4.76 (n=1)	
		2	0	4.76 (n=1)	
		3	0	0	
	3 (n=40)	0	100 (n=19)	90.47 (n=19)	0.386
		1	0	4.76 (n=1)	
		2	0	4.76 (n=1)	
		3	0	0	
	7 (n=40)	0	94.73 (n=18)	90.47 (n=19)	0.538
		1	5.26 (n=1)	9.52 (n=2)	
		2	0	0	
		3	0	0	
	14 (n=40)	0	94.73 (n=18)	90.47 (n=19)	0.538
		1	5.26 (n=1)	9.52 (n=2)	
		2	0	0	
		3	0	0	

Grades: 0= no symptom; 1 = relative change in sense of taste, 2 = no sense of taste

**Table 6:** Comparison of frequency of diarrhea and dizziness between the treatment and placebo groups on different days

**Factors**	**Time (day)**	**Grade**	**Treatment (%)**	**Placebo (%)**	**p-value (Chi-square test)**
**Frequency of diarrhea**	0 (n=40)	0	78.94 (n=15)	71.42 (n=15)	0.637
		1	15.78 (n=3)	14.28 (n=3)	
		2	5.26 (n=1)	14.28 (n=3)	
		3	0	0	
	3 (n=40)	0	84.21 (n=16)	85.71 (n=18)	0.543
		1	10.52 (n=2)	14.28 (n=3)	
		2	5.26 (n=1)	0	
		3	0	0	
	7 (n=40)	0	78.94 (n=15)	85.71 (n=18)	0.556
		1	15.78 (n=3)	14.28 (n=3)	
		2	5.26 (n=1)	0	
		3	0	0	
	14 (n=34)	0	94.73 (n=18)	100 (n=21)	0.475
		1	0	0	
		2	5.26 (n=1)	0	
		3	0	0	
Table 6. Continue					
**Frequency of dizziness**	0 (n=40)	0	52.63 (n=10)	57.14 (n=12)	0.913
		1	21.05 (n=4)	23.80 (n=5)	
		2	15.78 (n=3)	14.28 (n=3)	
		3	10.52 (n=2)	4.76 (n=1)	
	3 (n=40)	0	78.94 (n=15)	66.66 (n=14)	0.512
		1	21.05 (n=4)	28.57 (n=6)	
		2	0	4.76 (n=1)	
		3	0	0	
	7 (n=40)	0	89.47 (n=17)	85.71 (n=18)	0.380
		1	5.26 (n=1)	14.28 (n=3)	
		2	5.26 (n=1)	0	
		3	0	0	
	14 (n=34)	0	89.47 (n=17)	95.23 (n=20)	0.564
		1	5.26 (n=1)	4.76 (n=1)	
		2	5.26 (n=1)	0	
		3	0	0	

Grades: 0: no symptom; 1: mild; 2: moderate; 3: severe; 4: very severe

**Figure 3:**
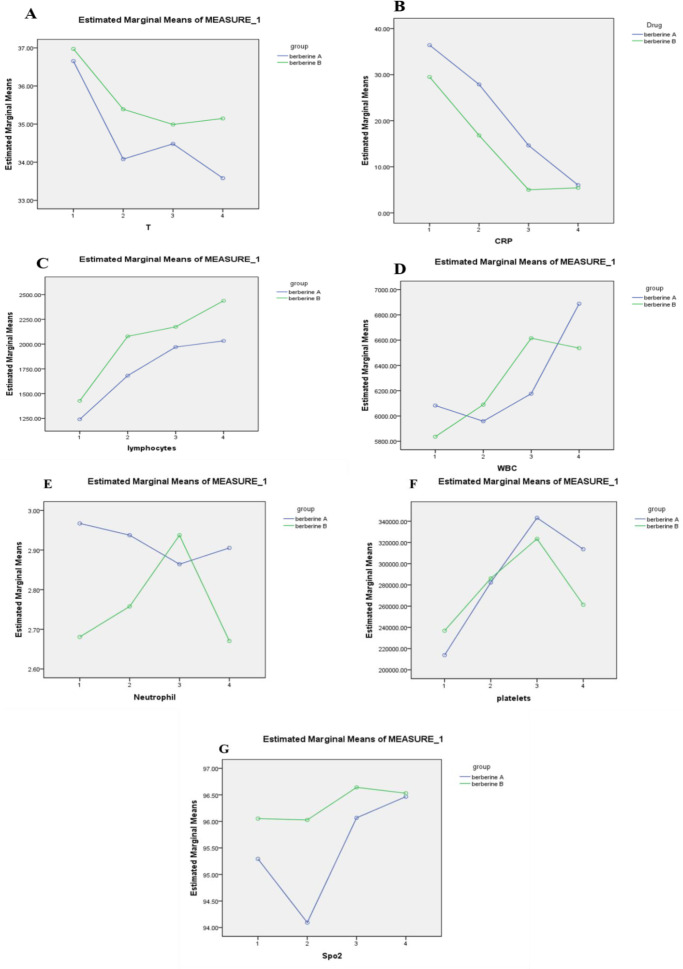
Comparison of A: body temperature; B: quantitative CRP level; C: WBC level; D: lymphocyte level; E: neutrophil level; F: platelet amount; G: and arterial oxygen level in the treatment and placebo groups on different days (Repeated measures test).

According to the obtained data, the body temperature on different days did not differ significantly between the two groups of treatment and placebo (p=0.259) (Figure 3A).

The intensity of body pain was not substantially different on different days (0, 3, 7, and 14) (p>0.05) between the treatment and placebo groups (Table 7).

Assessing the sore throat data illustrated that there was no significant alteration between the treatment and placebo group on days 0 (p=0.985), 3 (p=0.459), 7 (p=0.534), and 14 (p=0.293) (Table 7).

On days 0 (p=0.393), 3 (p=0.571), 7 (p=0.984), and 14 (p=0.132), the stomachache data revealed no significant differences between the treatment and placebo groups (Table 7).

**Table 7:** P values of body pain, sore throat, and stomachache between the treatment and placebo groups on different days

**Factors**	**Time (day)**	**P-value (Kruskal-Wallis)**
**Body pain**	0	0.945
	3	0.098
	7	0.116
	14	0.091
**Sore throat**	0	0.985
	3	0.459
	7	0.534
	14	0.293
**Stomachache**	0	0.393
	3	0.571
	7	0.984
	14	0.132

**Table 8:** Comparison of recovery times of quantitative CRP, lymphopenia, and clinical symptoms between the treatment and placebo groups

**Variable**	**Group**	**Mean±SD**	**p-value (Independent T-test)**
**Recovery time of quantitative CRP (days)**	Treatment	5.76±2.90	0.157
	Placebo	7.61±4.43	
**Recovery time of lymphopenia (days)**	Treatment	4.83±2.31	0.664
	Placebo	5.37±2.19	
**Recovery time of clinical symptoms (days)**	Treatment	10.05±4.23	0.426
	Placebo	11.15±4.13	

**Comparison of laboratory parameters between the treatment and placebo groups on different days**

Based on the obtained data, the amount of CRP was not significantly different between the two groups of treatment and placebo on different days (0, 3, 7, 14) (p=0.539) (Figure 3B).

The amount of WBC was not significantly different between the treatment and placebo groups on different days (p=0.825) (Figure 3C).

According to the results, the number of lymphocytes on different days did not differ significantly between the two groups of treatment and placebo (p=0.349) (Figure 3D).

The number of neutrophils on different days did not differ significantly between the two groups of treatment and placebo (p=0.922) (Figure 3E).

The number of platelets on different days did not differ significantly between the two groups of treatment and placebo (p=0.497) (Figure 3F).

According to the results, the arterial oxygen level on different days did not differ significantly between the two groups of treatment and placebo (p=0.725) (Figure 3G). 

**Comparison of recovery time of quantitative CRP, lymphopenia, and clinical symptoms**

According to the results, the duration of recovery of CRP in the treatment group was 5.76±2.90 days and in the placebo group was 7.61±4.43 days, which was not statistically significant (p=0.157) (Table 8).

The duration of recovery of lymphopenia daily in the treatment group was 4.83±2.31 days and in the placebo group was 5.37±2.19 days, which was not statistically significantly different (p=664).

The time interval for the improvement of clinical symptoms daily was 10.05±4.23 days in the treatment group and 11.15±4.13 days in the placebo group (p=0.426).

**Adverse events**

Some people experienced nausea and diarrhea after using berberine capsules. 

## Discussion

The data obtained from the present clinical trial demonstrated that berberine (300 mg, TID for 14 days) had no significant effect on clinical symptom improvement (cough, shortness of breath, nausea, loss of smell and taste, runny or stuffy nose, diarrhea, dizziness, sore throat, stomachache, body aches, and body temperature), quantitative CRP, lymphopenia, WBC, lymphocytes, neutrophils, platelets, and SpO_2_.

COVID-19 has a wide spectrum of clinical manifestations, ranging from asymptomatic infections to life-threatening illnesses. Cough, dyspnea, malaise, fatigue, and fever are the most prevalent symptoms of COVID-19, which are similar to the symptoms of a viral infection and pneumonia (Singhal, 2020). In the present study, patients were questioned about various clinical symptoms and their severity according to associated criteria at the start of the study as well as on days 3, 7, and 14 after starting the medication. Based on the data, berberine was found to not affect clinical manifestations of COVID-19.

The treatment of viral diseases is always difficult. Modulating the immune system is one of the most effective ways to combat viral infections (Kikkert, 2020). An aberrant leukocyte count has been identified in COVID-19 disease, which leads to immune system suppression (Jesenak et al., 2020). This is a viral escape mechanism that prevents the virus from replicating in human cells (Shah et al., 2020). Berberine (100 mg, TID for 14 days) had no significant effect on WBC count. In line with our results, it was observed that the administration of berberine (900 mg, daily, 14 days) to patients with severe COVID-19 did not affect WBC count (Zhang et al., 2021).

According to the guidelines, CRP, an inflammatory marker, must be assessed in COVID-19 patients (Smilowitz et al., 2021). In the liver, IL-6 produces this marker and it is referred to as an "acute phase reactant" (Potempa et al., 2020). In COVID-19 disease, the level of CRP rises. The severity of the disease is determined by the amount of CRP (Wang, 2020). CRP levels were high in both the placebo and berberine groups at the start of the study. CRP was lowered more in the berberine group than in the placebo group, but it was not significant. This decline has continued for 14 days. It was observed that berberine supplementation could reduce CRP amount in patients with metabolic disorders (Asbaghi et al., 2020; Imenshahidi and Hosseinzadeh, 2019). But, the prescription of berberine (900 mg, daily, 14 days) to patients with severe COVID-19 could not affect CRP levels (Zhang et al., 2021). This contradiction might be explained by the higher doses of berberine administered to patients with metabolic disorders and the different underlying mechanisms involved in these disorders. 

Berberine has direct anti-influenza virus activities *in vitro*, suppresses lung inflammatory injury, and lowers the generation of oxygen radicals in mice with influenza-related pneumonia (Liu et al., 2020; Wu et al., 2011). In addition, since a clinical trial reported that zinc oxide nanoparticle/berberine acts as an anti-COVID-19 (Ghareeb et al., 2021), it might be suggested that nanoparticles and employing an appropriate pharmaceutical formulation might increase the therapeutic properties of berberine in managing coronavirus. Further investigations are required to determine the optimum berberine dose for inflammation related to COVID-19. 

It is necessary to note that because inpatients were not included in our trial, our team was unable to explore the effect of berberine on severe COVID-19 illness or fatality rates. In general, it is suggested that future investigations be carried out on larger sample size for a longer time with higher doses and appropriate pharmaceutical formulations.

**Limitations, strengths, and weaknesses**

-Uncertainty about patients' total adherence to the treatment regimen. 

- Lack of cooperation on the part of some patients in answering related questions and undertaking tests. 

- Small research sample size

As per our study findings, berberine (300 mg, three times a day, 2 weeks) does not have any positive effect on controlling and treating COVID-19 but because of the limitations of our study, the results cannot be generalized. 

## Conflicts of interest

The authors have declared that there is no conflict of interest.
